# Supplementary material for: Cardiac Magnetic Resonance in Patients with Suspected Tachycardia-Induced Cardiomyopathy: The Impact of Late Gadolinium Enhancement and Epicardial Fat Tissue
Source: J Pers Med. 2023 Sep 27;13(10):1440. doi: 10.3390/jpm13101440 (PMC10607955; doi:10.3390/jpm13101440)
Supplement: Supplementary file 1 [file jpm-13-01440-s001.zip › jpm-2511479-supplementary.pdf]

Supplementary image

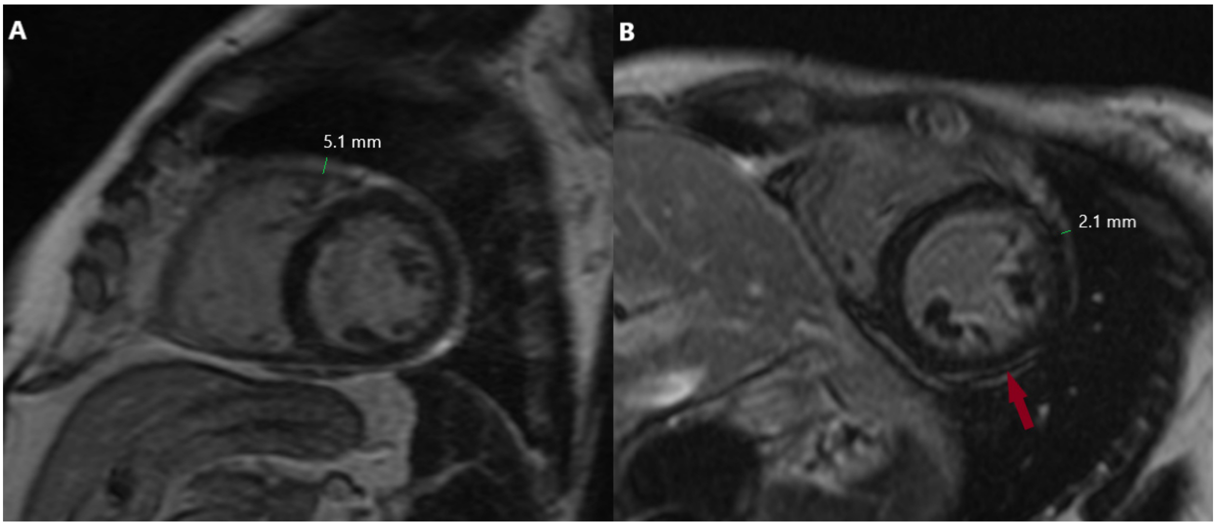

Figure S1 Cardiac MRI in responders (A) and in non-responders (B), LGE sequences.

A. MRI demonstrate a lack of LGE in responders, thickness of epicardium fat (green line) 5.1 mm
B. MRI shows intramyocardial LGE (red arrow) in the inferolateral wall in non-responders, thickness of epicardium fat (green line) 2.1 mm

Supplementary table S1. Cardiac MRI values for patients with sinus rhythm and AF.

	**Patients with sinus rhythm (n=20)**	**Patients with AF (n=9)**	**P-value**
Age	58.2 ± 18.7	58.1 ±13.24	0.5
Male. n (%)	10 (50)	9 (100)	0.01
Charlson comorbidity index. points	3 [1;6]	4 [1:7]	0.17
LVEDV. ml	80.6 ±34.2	112.8 ±44.7	0.5
RVEDV. ml	66 ±14.3	105.6 ±44.3	0.24
LVEF at admission (TTE). %	37.3 ±9.8	36.3 ±7.8	0.67

LVEF in 14 days after DC (TTE). %	50.3 ±8.8	42.6 ±13.4	1.0
Responders. n (%)	15 (75)	2 (22.2)	0.03
RVEDV CMR. ml/m2	66.1±6.7	105.7±34.1	0.001
LVEDV CMR. ml/m2	80.7±16.0	112.9±35.1	0.04
LA long axis cmr/m2	2.79 ±0.5	3.6 ±0.89	0.5
LA short axis cmr/m2	2.19 ±0.46	2.8 ±0.66	0.048
RA long axis cmr/m2	2.67 ±0.49	3.44 ±0.79	0.01
RA short axis cmr/m2	2.37 ±0.48	2.9 ±0.6	0.03
IVS mm. cmr	10.4 ±2.6	10.2 ±1.2	1.0
Patients with LGE. n (%)	8 (40)	4 (44.4)	0.56
% LGE in whole group	2.48 ±4.7	2.74 ±3.42	1.0
Heart rate (average; range)	67.5 [52-82]	83.3 [62-115]	0,04
Interobserver variability in LGE identification	10%	0%	

LVEDV - left ventricular end-diastolic volume. RVEDV - right ventricular end-diastolic volume. LVEF – left ventricular ejection fraction. TTE - transthoracic echocardiogram. LA – left atrium. RA – right atrium. IVS - interventricular septum. LGE - late gadolinium enhancement
